# Binding and neutralization of *C*. *difficile* toxins A and B by purified clinoptilolite-tuff

**DOI:** 10.1371/journal.pone.0252211

**Published:** 2021-05-27

**Authors:** Carmen Ranftler, Dietmar Nagl, Andreas Sparer, Andreas Röhrich, Michael Freissmuth, Ali El-Kasaby, Shahrooz Nasrollahi Shirazi, Florian Koban, Cornelius Tschegg, Stephane Nizet

**Affiliations:** 1 GLOCK Health, Science and Research G.m.b.H., Deutsch-Wagram, Austria; 2 Institute of Pharmacology & Gaston H. Glock Research Laboratories for Explorative Drug Development, Centre of Physiology and Pharmacology, Medical University of Vienna, Vienna, Austria; University of Illinois at Chicago, UNITED STATES

## Abstract

*Clostridioides difficile (C*. *difficile)* infection is a major public health problem worldwide. The current treatment of *C*. *difficile*-associated diarrhea relies on the use of antibacterial agents. However, recurrences are frequent. The main virulence factors of *C*. *difficile* are two secreted cytotoxic proteins toxin A and toxin B. Alternative research exploring toxin binding by resins found a reduced rate of recurrence by administration of tolevamer. Hence, binding of exotoxins may be useful in preventing a relapse provided that the adsorbent is innocuous. Here, we examined the toxin binding capacity of G-PUR®, a purified version of natural clinoptilolite-tuff. Our observations showed that the purified clinoptilolite-tuff adsorbed clinically relevant amounts of *C*. *difficile* toxins A and B *in vitro* and neutralized their action in a Caco-2 intestinal model. This conclusion is based on four independent sets of findings: G-PUR® abrogated toxin-induced (i) RAC1 glucosylation, (ii) redistribution of occludin, (iii) rarefaction of the brush border as visualized by scanning electron microscopy and (iv) breakdown of the epithelial barrier recorded by transepithelial electrical resistance monitoring. Finally, we confirmed that the epithelial monolayer tolerated G-PUR® over a wide range of particle densities. Our findings justify the further exploration of purified clinoptilolite-tuff as a safe agent in the treatment and/or prevention of *C*. *difficile*-associated diarrhea.

## Introduction

Over the past three decades, *Clostridioides difficile* (*C*. *difficile*), previously known as *Clostridium difficile*, a gram positive, anaerobic, spore-forming bacillus has emerged as a major nosocomial pathogen [1, 2]. In addition, a substantial fraction of *C*. *difficile*-associated diseases (CDAD) are community-acquired [3]. In a recent report, the Center for Disease Control and Prevention (CDC) estimated that, annually, there were about 500,000 *C*. *difficile* infections (CDI) and close to 30,000 C. *difficile*-related deaths in the USA [4]. A Europe-wide coordinated surveillance program, which was initiated by the European Centre for Disease Prevention and Control (EDC) in 2016, provided a similar estimate and indicated that about 4% of CDI resulted in a fatal outcome [5].

The main virulence factors of *C*. *difficile* are two cytotoxic proteins, referred to as toxin A and toxin B. Their mechanism of action is understood in considerable detail [6]: they are toxins composed of modular domains which work synergistically to bind membrane receptors on the apical surface of the intestinal epithelium [7, 8]. Endocytosis of toxin-receptor complexes can be clathrin-dependent or independent [9, 10]. The acidification of the endosome triggers the insertion of hydrophobic segments into the endosomal membrane, which allows for translocation of the N-terminal portion into the cytosol [11]. Activation takes place by an autocatalytic process activated by cytosolic inositol hexakisphosphate [12, 13]. This releases the adjacent N-terminal glucosyltransferase domain, which is in turn recruited to the inner leaflet of the cell membrane via a four helix bundle, where it monoglucosylates monomeric G proteins of the Rho family, including RHO-A, -B, -C, RAC1 and CDC42, by using cellular UDP-glucose as co- substrate [6, 14]. Glucosylation of a conserved threonine in the switch-I region of the G proteins blocks their binding to effectors [6]. This results in the disorganization of the F-actin network and the loosening of the tight junctions [15–17]. The disruption of the intestinal barrier causes diarrhea, which can elicit symptoms ranging from mild water loss to acute, life-threatening pseudomembranous enterocolitis. In addition, glucosylated Rho proteins are recognized by pyrin, which triggers the assembly of the inflammasome and the activation of caspase-1 [18]. The subsequent stimulation of interleukin-8 secretion drives neutrophil infiltration, which contributes to the development of pseudomembranous enterocolitis [19].

Because *C*. *difficile*-associated disease (CDAD) is due to bacterial toxins secreted into the gut lumen, the use of toxin binders has been repeatedly advocated. In fact, the anion exchange resins cholestyramine and colestipol were tested some forty years ago [20, 21]. However, they have proven ineffective in randomized controlled trial [22, 23]. Tolevamer, an anionic polymer specifically developed to neutralize C. *difficile* toxins [24], was inferior to antibacterial therapy with vancomycin or metronidazole in terminating diarrhea; however, the rate of recurrence was lower in tolevamer-treated patients [25]. This observation justifies the search for improved toxin-binders. Recently, *C*. *difficile* toxins A and B were shown to bind to a calcium aluminosilicate *in vitro* [26]. Other minerals, such as zeolites, have a remarkable capacity to bind various chemical and biological compounds [27]. For our study, G-PUR®, a purified and micronized natural zeolite-tuff that was processed through a patented, thoroughly qualified and quality-assured manufacturing procedure to optimize its safety and efficiency, was used [28]. G-PUR® is mainly composed of the aluminosilicate clinoptilolite, a natural zeolite mineral with peculiar binding affinity to medically relevant substances [27]. Among other characteristics, clinoptilolite has been shown to bind and neutralize mycotoxins, a property that warrants its use in animal husbandry [29].

Here, we examined the ability of G-PUR® to adsorb *C*. *difficile* toxins A and B *in vitro*. We verified that the adsorptive capacity of G-PUR® sufficed to neutralize the effects of *C*. *difficile* toxins A and B on intestinal epithelial cells *in vitro*. Caco-2 cell monolayers recapitulate some functional and morphological features of mature enterocytes, like basolateral and apical polarization with vectorial transport of solutes, formation of the typical brush border and of tight junctions, which afford a high transepithelial electrical resistance [30–32]. When challenged with clostridial toxins, enterocytes undergo morphological changes in their actin cytoskeleton resulting in loss of tight junctions and microvilli and a breakdown of their barrier function [33, 34]. Our experiments showed that G-PUR® preserved the integrity of the Caco-2 cell monolayers and obviated these effects of *C*. *difficile* toxins A and B.

## Materials and methods

### Materials

Stock solutions of *C*. *difficile* toxins A and B (The Native Antigen Company, Oxford, UK) were reconstituted at a concentration of 200 μg/ml following the manufacturer’s instructions. The working solution was obtained by first diluting each aliquot to 2 μg/ml with DMEM-based growth medium supplemented with 10% fetal calf serum (FCS, M&B Stricker, Tutzing, Germany). Thereafter, both toxin A and B stock solutions were mixed together in a 1:1 ratio. Appropriate further dilutions were made with DMEM-based growth medium supplemented with 0.1% FCS. The zeolite material G-PUR® (manufactured by Glock Health, Science and Research G.m.b.H., Austria) used in this study is a purified micronized clinoptilolite-tuff, produced from a high-grade raw material, mined in the eastern Slovak Republic [28, 35]. The purification process is technically based on ion exchange mechanisms of the clinoptilolite-tuff, micronization and terminal heating, which results in the removal of all natural impurities and a homogeneous particle size. The production process is thoroughly quality-assured, meeting all required regulatory standards. Buffer salts and reagents were analytical grade or of the highest commercially available quality.

### Cell culture

Caco-2 cells (ATCC HTB-37) were grown in Dulbecco’s Modified Eagle’s Medium (VWR, Vienna, Austria) supplemented with 10% FCS, 2 mM glutamine (VWR, Vienna, Austria), 50 mg/l each penicillin and streptomycin (VWR, Vienna, Austria) and 1% non-essential amino-acids (VWR, Vienna, Austria). Cells were seeded onto 24-well Transwell™ membrane inserts (Corning, USA) placed in 24-well companion plates (Corning, USA), 24-well plates (for resazurin assay) or 3 cm Ø dishes (for glucosylation assay) and grown until day 21 after confluence to form a monolayer of polarized epithelial cells. In all incubation solutions containing clostridial toxins, the FCS concentration was lowered to 0.1%.

### Binding of *C*. *difficile* toxins to G-PUR®

The *in vitro* binding of toxins to G-PUR® was determined by adding the solution of *C*. *difficile* toxins A & B (1:1) at different concentrations ranging from 5 to 60 ng/ml to 5 ml tubes (Eppendorf, Wesseling-Berzdorf, Germany), containing 16 mg G-PUR® powder in a final volume of 4 ml. The microtubes were further incubated on a rotator (Stuart, UK) at 37°C for the indicated time intervals. The incubation was terminated by centrifugation for 3 min at 19,000 x g to pellet the G-PUR® particles. The clostridial toxins were quantified in the supernatant using the Ridascreen *C*. *difficile* Toxin A/B ELISA test kit (R-Biopharm, Darmstadt, Germany) according to the manufacturer’s instructions. The limit of detection was 0.2 ng/ml. The standard curve was linear up to 5 ng/ml. Samples were diluted appropriately to fall within the linear range of the standard curve. Two independent kinetic experiments (each done in quadruplicate, the means of which are shown) were carried out to determine the time course of toxin binding to G-PUR®. The resulting data points for disappearence of toxins from the solution were subjected to non-linear least-squares curve fitting to the equation for a double-exponential decay using a Marquardt-Levenberg algorithm. Saturation binding was determined in three independent experiments (each done in quadruplicate, the means of which are shown). The resulting data were subjected to non-linear least-squares curve fitting to the three-parameter logistic equation (Hill equation). We report the extracted parameter estimates (i.e. the dissociation constant K_D_ and the maximum binding B_max_) and their standard errors. Three experiments (each done in triplicate) were carried out to examine the effect of 0.24 mM (= 0.01%) and 2.4 mM sodium deoxycholate (= 0.1%) or of 0.01% and 0.1% bile salts (Sigma Aldrich, St. Louis, MO, USA) on the binding of C. *difficile* toxins to G-PUR®. These data are shown as means ± S.D. We verified that the presence of deoxycholate and bile salts at the concentrations tested did not interfere with the quantification method of C. *difficile* toxins.

### Binding of radiolabeled sodium deoxycholate to G-PUR®

#### Binding of radiolabeled sodium deoxycholate to G-PUR®

Binding of [^3^H]deoxycholate (ARC, American Radiochemical Company; 22 Ci/mmol) was measured in a final volume of 0.5 ml containing synthetic intestinal fluid (50 mM KH_2_PO_4_, pH adjusted to 6.8 with NaOH according to the international Pharmacopeia), 10 mg G-PUR® (preequilibrated in synthetic intestinal fluid) and the concentrations of unlabeled deoxycholate ranging from 1 μM to 3 mM resulting in a specific activity ranging from about 100 cpm/pmol to 30 cpm/nmol. The suspension was incubated for 2 h by end-over-end rotation; thereafter, bound and free radioactivity were separated by centrifugation (5 min at 13,000 x g). The radioactivity in an aliquot of the supernatant (0.25 ml) was determined by liquid scintillation counting. Three independent experiments (each in duplicate) were carried out and the data are shown as means ± S.D. The data were subjected to non-linear least-squares curve fitting to the equation for a rectangular hyperbolato extract parameter estimates (i.e. the dissociation constant K_D_ and the maximum binding B_max_) and their standard errors.

### Incubation of Caco-2 cells with *C*. *difficile* toxins A and B, which had or had not been pre-adsorbed to G-PUR®

G-PUR® (80 mg) was dispensed in powder form into 15 ml tubes (TPP, Trasadingen, Switzerland). Cell culture medium containing 0.1% FCS with and without *C*. *difficile* toxins A & B (1:1) was added for a total volume of 6 ml. The powder was briefly resuspended by vortexing and subsequently sonicated for 5 min at 37°C in a Sonorex sonicator bath (Bandolin, Berlin, Germany). Thereafter, samples were incubated for additional 10 min at 37°C. After centrifugation at 19,000 x g for 3 min, the resulting supernatant was transferred to the Caco-2 cell monolayer grown on Transwell™ inserts and incubated for up to 24 h at 37°C. The cells were also exposed in parallel to cell culture medium containing 0.1% FCS with and without clostridial toxins A & B (1:1), which had not been pre-incubated with G-PUR®. At the end of the incubation time, the cells were rinsed and fresh medium containing 0.1% FCS was added. The cells were either fixed (for occludin staining and scanning electron microscopy) or used for resistance measurements.

### Viability assay and flow cytometry

The effect of G-PUR® on cell viability was measured by flow cytometry using propidium iodide labeling and by quantifying the ability of the cells to reduce resazurin to resorufin, which can be measured by fluorometry. The stock solution of resazurin (1 mM) was prepared in phosphate-buffered saline (PBS) (Sigma Aldrich, St. Louis, MO, USA), filter-sterilized and stored at room temperature in the dark. Working solutions were prepared by diluting the stock solution 100-fold with growth medium containing 10% FCS. For the assay, polarized Caco-2 cells (21 days post-confluence) were incubated for 3 h with the working solution in 24-well dishes. Readings were performed with a Biotek Synergy HT instrument (Biotek, Bad Friedrichshall, Germany) in fluorescence mode at an excitation wavelength of 530 nm and an emission wavelength of 590 nm. Background fluorescence, measured in growth medium supplemented with 10 μM resazurin and incubated without cells, was subtracted from each sample measurement. Measurements are expressed as relative fluorescence units (RFU). Four independent experiments (each done in quadrupicate) were carried out. The data are reported descriptively as means ± S.D..

For flow cytometry, polarized Caco-2 cells, which had been incubated in the absence and presence of G-PUR®, were detached with TrypLE (Thermo Fisher Scientific, Walham, MA, USA), centrifuged for 3 min at 100 x g and suspended in DMEM medium without FCS. Cells were subsequently stained with 10 μM propidium iodide (PI, Sigma Aldrich, St. Louis, MO, USA) for 5 min at room temperature. Readings were performed with a Cube8 flow cytometer (Sysmex, Vienna, Austria) at wavelengths of 538 nm for the excitation and 617 nm for the emission. In each sample, the analysis is based on a minimum of 23,000 cells. Negative and positive controls for PI staining were prepared using either viable Caco-2 cells, which had been stained with 5-carboxyfluorescein diacetate (Sigma Aldrich, St. Louis, MO, USA), or Caco-2 cells, which had been killed by heating at 90°C for 10 min. The data from three independent experiments are reported descriptively as means ± S.D.

### Determination of RAC1 glucosylation

Confluent Caco-2 monolayers were incubated in medium containing 0.1% FCS in the absence and presence of *C*. *difficile* toxins A and B (0.2 ng/ml, equimolar mixture) for 20 h at 37°C. After the incubation, cells were detached and lysed in a buffer composed of 50 mM Tris-HCl (pH 7.5), 1 mM EDTA, 2 mM CaCl_2_ and 1 mM DTT. The samples were snap-frozen in liquid nitrogen and stored at -80°C until further use. After rapid thawing, the suspension was extracted in buffer containing 50 mM Tris-HCl (pH 8.0), 150 mM NaCl, 1% dodecylmaltoside, 1 mM EDTA, and Complete™ protease inhibitor mixture (Roche, Austria); the lysates were rotated at 4°C for 1 h. Insoluble material was removed by centrifugation (30 min at 13,000 x g at 4°C). Aliquots from the lysates (40 μg) were resolved by SDS-polyacrylamide gel electrophoresis (monomer concentration 13%). Proteins were transferred onto nitrocellulose membranes, which were blocked with 3% nonfat dry milk in PBS. Membranes were incubated with a murine monoclonal antibody recognizing glucosylated and non-glucosylated forms of RAC1 (clone 23A8; Cat. No. 05–389; Merck Millipore, Darmstadt, Germany) or a murine monoclonal antibody, which detects only the non-glucosylated form of RAC1 (clone 102; Cat. No. 610651; BD Biosciences, Franklin Lakes, NJ, USA) at a dilution of 1:1000 in 3% nonfat dry milk in PBS overnight at 4°C [36]. Subsequently, membranes were washed in PBS containing 0.05% Tween 20, and incubated with a horseradish peroxidase-(HRP)-conjugated, anti-mouse IgG secondary antibody (1: 5,000) for 1 h at room temperature. Immunodetection of G protein β-subunits (Gβ) by an affinity purified rabbit antiserum directed against the N-terminus of Gβ1 and Gβ2 was done as a loading control [37]. The immunoreactivity was detected by chemiluminescence (FluorChem HD2 system, Alpha Innotech, San Jose, CA, USA). The color of the resulting images was inverted with Image J in order to obtain dark bands on a white background. The quantification of band intensity was done by densitometry using the built-in software. Three independent experiments were carried out and the ratio of non-glucosylated RAC1 signal over total RAC1 signal was quantified (individual values are shown in the pertinent figure together with median and interquartile range). Because the data passed the Shapiro-Wilk normality test, the statistical comparison was done by repeated measures ANOVA followed by all possible pairwise comparisons using the Holm-Sidak approach with a threshold of significance at p<0.05.

### Analysis of the apical surface of Caco-2 cells by scanning electron microscopy

The confluent Caco-2 cell monolayers grown on Transwell™ inserts were fixed for 1 hour at room temperature by incubation in a solution containing 2.5% glutaraldehyde and 2% paraformaldehyde in 50 mM HEPES (pH 7.2). After washing with MilliQ-purified water, the samples were dehydrated in a graded ethanol series (0, 25, 50, 70, 95 and 100%; VWR, Vienna, Austria) and incubated twice for 5 min with hexamethyldisilazane (VWR, Vienna, Austria) at room temperature. After removal of the solution, the samples were left to dry over night at room temperature. On the next day, the dried Transwell™ membranes were excised from the support, mounted onto aluminum stubs (Science Services, Munich, Germany) and gold-sputtered at 3 different angles (Cressington Sputter Coater 108 auto, Cressington Scientific Instruments Ltd., Watford, UK). Samples were examined with a Tescan Vega series scanning electron microscope (Brno, Czech Republic) operated at 20 kV. Two insert membranes were analyzed per treatment out of two independent experiments. For each sample, 3 overview images were taken at low magnification (approximately 3,000-fold; representing an area of 100x80μm; corresponding to 80,000μm² each) and out of each overview picture at detailed view was taken at a higher magnification (13,000–20,000-fold).

### Immunofluorescence staining of occludin

The confluent Caco-2 cell layers grown on Transwell™ inserts were subjected to incubations in medium (control condition), medium pre-adsorbed for 15 min to G-PUR® (4 mg/ml), medium containing an equimolar mixture of *C*. *difficile* toxins (2.2 ng/ml, equimolar mixture) or medium containing *C*. *difficile* toxins preadsorbed to G-PUR® (4 mg/ml) for 24 h. Thereafter, the medium was removed and the cells were incubated for 20 min at room temperature with a fixation solution containing 2% paraformaldehyde in 50 mM HEPES (pH 7.2). After fixation, the Transwell™ membranes were excised with a scalpel and the cells were permeabilized for 10 min at room temperature using 0.1% Triton X-100 (Sigma Aldrich, Austria) in PBS. Prior to addition of the antibodies, non-specific binding sites were blocked by an incubation in the presence of 5% FCS in PBS for 30 min at room temperature. Thereafter, the samples were incubated with an antigen affinity purified rabbit polyclonal antibody directed against occludin (ProteinTech, USA No. 13409-1-AP) at a 1:50 dilution in PBS containing 5% FCS for 1.5 h at 37°C. After three washes with PBS, the secondary Cy3-labeled goat anti-rabbit antibody (No. SA00009-2, ProteinTech, Rosemont, IL, USA) was added at a 1:100 dilution for 1.5 h at 37°C. Finally, the samples were mounted onto a cover slip with the antifading agent Vectashield (Vecto Laboratories, USA). Immunostaining of the cells was imaged using a 60x oil immersion objective on a Nikon A1+ confocal laser-scanning microscope (Nikon, Minato, Japan) equipped with a diode laser (at 561 nm and 30 mW). First, samples were scanned along the z-axis to determine the distribution of occludin related to the depth of the optical sectioning. Subsequently, images were captured in the subapical region, where occudin staining was of maximum intensity. To quantify the redistribution of occludin, the digital camera was calibrated and a fluorescence profile crossing the membrane staining at an angle of 90° was drawn. The width of the membrane staining was determined using the Zen software provided by the microscope manufacturer. A minimum of 50 randomly-selected cells per condition was used. The statistical comparison was done by a Kruskal-Wallis test followed by Dunn’s pairwise assessment of all possible comparisons. Two independent experiments were carried out and twenty visual fields were examined in each sample using identical settings (pinhole diameter, laser intensity, dwell time).

### Recordings of transepithelial electrical resistance

The transepithelial electrical resistance (TEER) [38] was recorded using a Millicell ERS-2 Volt-Ohmmeter (Merck Millipore, Darmstadt, Germany) by delivering rectangular alternating currents (10 μA) through chopstick electrodes at 12.5 Hz according to the instructions of the manufacturer. Caco-2 cell monolayers grown on Transwell™ inserts were incubated in medium (control condition), medium pre-adsorbed for 15 min to G-PUR® (4 mg/ml), medium containing an equimolar mixture of *C*. *difficile* toxins (2.2 ng/ml, equimolar mixture) or medium containing *C*. *difficile* toxins pre-adsorbed to G-PUR® (4 mg/ml). At the indicated time points in the pertinent figure (0, 2, 3, 4, 5, 6, 7 and 24 h), the medium was replaced by fresh medium and the transepithelial electrical resistance (TEER) was measured. Because TEER is dependent on temperature, the cellular monolayers were kept at 37°C using a thermomixer Comfort 5355 (Eppendorf, Wesseling-Berzdorf, Germany) during all measurements. The recorded resistance was multiplied by the surface area (Ωcm²). Three independent experiments (each in sextuplicate) were carried out. The data are reported as means ± S.D. from these three determinations. Because the data passed the Shapiro-Wilk normality test, the statistical comparison was done by repeated measures ANOVA followed by all possible pairwise comparisons using the Holm-Sidak approach with a threshold of significance at p<0.05.

## Results

### Viability of Caco-2 cells in the presence of G-PUR®

**Fig 1:**
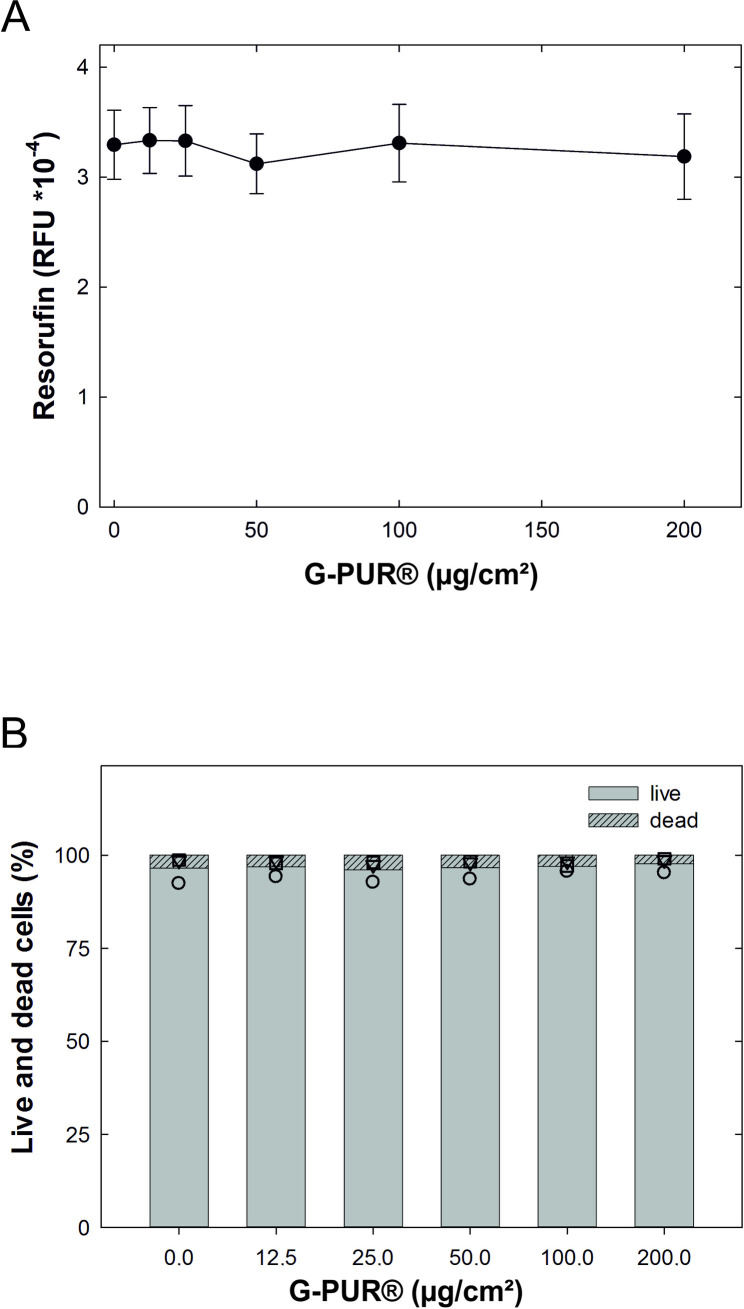
Effect of G-PUR® treatment on the viability of Caco-2 cells after 24h incubation. A. Increasing concentrations of G-PUR® had not effect on the reduction of resazurin by Caco-2 cells. B. Flow cytometry after propidium iodide staining showed no increase in the dead:live ratio of Caco-2 cells.

When added to cell cultures, clinoptilolite has been claimed to affect several intracellular signaling pathways, which inhibit cell growth and reduce cell viability [39]. Accordingly, we first determined, if G-PUR® affected the viability of Caco-2 cells *in vitro*. Caco-2 cells are derived from a human adenocarcinoma of the colon, which undergo spontaneous differentiation post-confluence, forming monolayers with apical and basolateral membrane compartments, microvilli and tight junctions and thus share many characteristics of enterocytes of the human small intestine [30]. We exposed monolayers of polarized Caco-2 cells to increasing amounts of G-PUR® for 24 h. Because of their density, G-PUR® particles rapidly sediment onto the cell layer. Hence, we expressed the concentration of G-PUR® as particle weight/area unit (μg/cm^2^) rather than as their concentration in the medium. Over the entire range tested (12.5 to 200 μg/cm^2^), G-PUR® did not affect cell viability based on the ability of cells to reduce resazurin (Fig 1A). We also assessed the proportion of dead cells by flow cytometry after propidium iodide staining: it is evident from Fig 1B that–regardless of the presence or absence of G-PUR® –over 95% of Caco-2 cells excluded propidium iodide. Thus, based on two independent approaches, we concluded that G-PUR® did not alter cell viability.

### Binding of *C*. *difficile* toxins A and B to G-PUR®

G-PUR® binds mycotoxins and this feature is one of the rationales underlying its use as additive in animal feed [29, 40]. We examined if this was also true for the large *C*. *difficile* toxins. An equimolar mixture of *C*. *difficile* toxins A and B was incubated with G-PUR®, the particles were subsequently removed by centrifugation and the toxins remaining in solution were quantified in the supernatant by ELISA.

**Fig 2:**
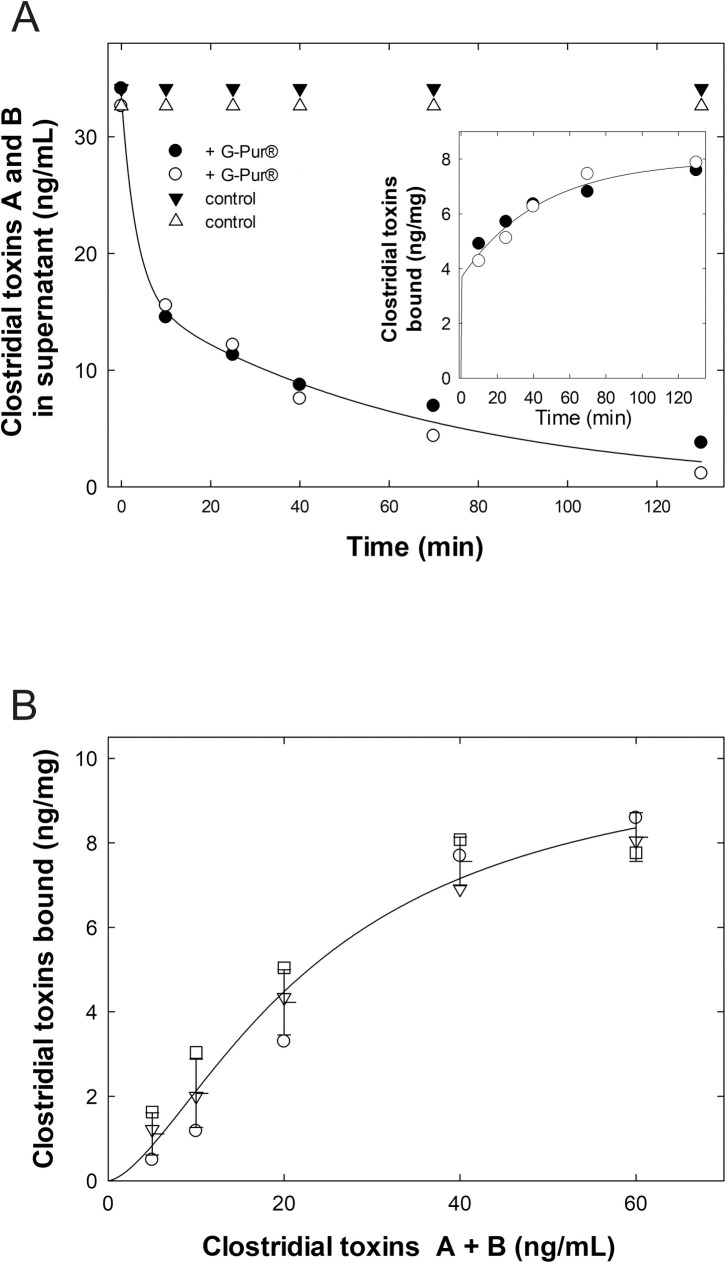
Time course of binding (a) and saturation binding (b) of clostridial toxins to G-PUR®. A: An equimolar mixture of *Clostridioides difficile* toxins A and B was incubated in the absence (triangles, control) and presence of 4 mg/ml G-PUR® (circles) for increasing incubation times. Data represent 2 independent experiments carried out in quadruplicate. Full and empty symbols refer to the first and the second experiment, respectively. After centrifugation, the clostridial toxins in the supernatant were quantified by ELISA. The solid line was drawn by fitting the data to the equation for a double-exponential decay (rate constants for the first and the second phase = 0.30 ± 0.15 min^-1^ and 0.016 ± 0.002 min^-1^, respectively). The insert shows the bound clostridial toxins (ng per mg G-PUR®) calculated from the difference measured in the absence and presence of G-PUR®. B: A constant amount of G-PUR® (4 mg/ml) was added to the indicated concentration of *Clostridioides difficile* toxins A and B (1:1 mixture). The suspension was incubated for 2 h at 37°C. Data are from 3 independent experiments carried out in quadruplicate. Each individual experiment is shown as a distinct symbol; means and error bars (S.D.) re also shown. The data were subjected to non-linear, least-squares curve fitting to the three-parameter logistic equation (Hill-equation) to yield estimates for K_D_ = 23.7 ± 7.0 ng/ml and B_max_ = 10.3 ± 1.8 ng/mg and to draw the solid line.

Binding of the toxins was biphasic. Accordingly, the data were best described by a biexponential decay (Fig 2A): during the first 10 minutes of the incubation, the concentration of *C*. *difficile* toxins in the supernatant dropped rapidly (rate constant of the first phase = 0.30 ± 0.15 min^-1^). Thereafter, their concentration decreased at a slower rate (rate constant of the second phase = 0.016 ± 0.002 min^-1^). The amount of bound toxins was calculated for each time point as the difference between the control incubation (Fig 2A) and the incubation done in the presence of G-PUR® (Fig 2A). This yielded an association curve, which reached a plateau after 120 minutes (Fig 2A, insert). Based on the time course shown in Fig 2A, increasing concentrations of an equimolar mix of toxins A and B were incubated for 2 hours at 37°C with a constant G-PUR® concentration of 4 mg/ml. This concentration was based on the recommended intake of 2 g of G-PUR® by the manufacturer and a stomach volume of 500 ml. The remaining unbound toxins were again quantified in the supernatant to calculate the fraction absorbed by G-PUR®. Based on the resulting saturation curve, a K_D_ of 23.7 ±7.0 ng/ml and a maximal binding capacity of 10.3 ± 1.8 ng/mg were calculated (Fig 2B).

**Fig 3:**
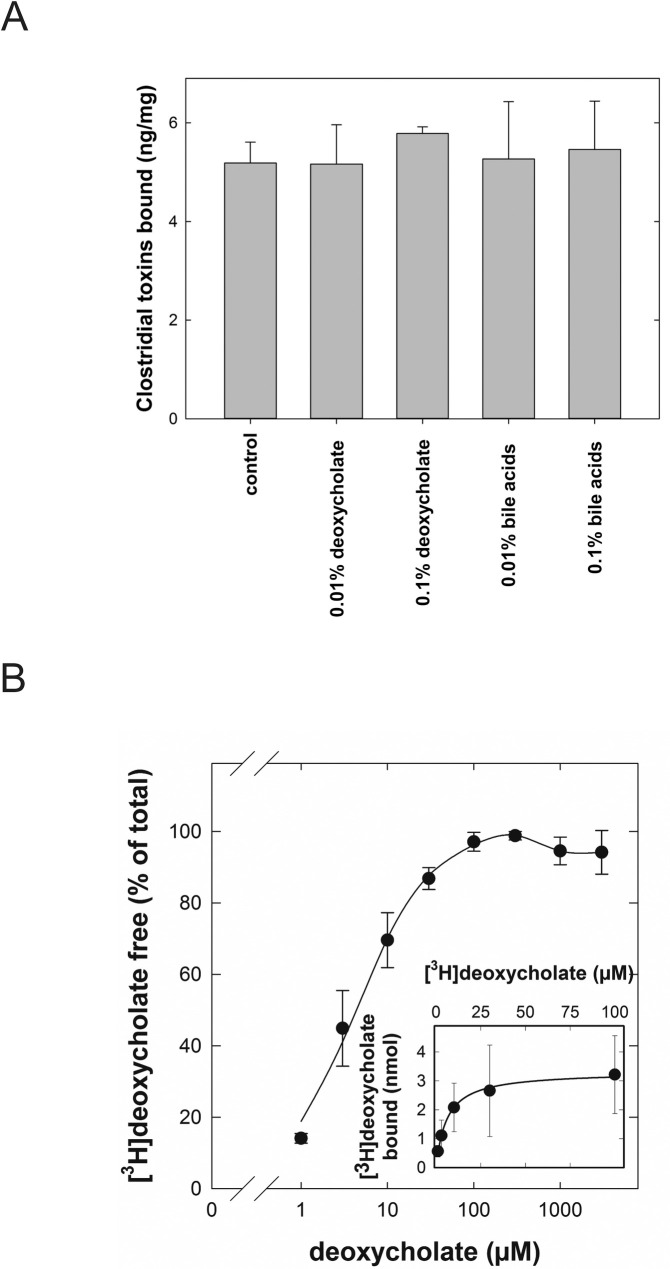
Interaction of G-PUR® with sodium deoxycholate and its ability to bind C. *difficile* toxins. A: An equimolar mixture of *Clostridioides difficile* toxins A and B was incubated with G-PUR® in the presence or absence of 0.1% (2.4 mM) and 0.01% (0.24 mM) deoxycholate or of bile salts. After incubation at 37°C, G-PUR® was pelleted by centrifugation and the concentration of clostridial toxins remaining in the supernatant was determined by ELISA. Data from 3 independent experiments carried out in triplicate are displayed as means ± standard deviations. B: [^3^H]deoxycholate was incubated at different concentrations in synthetic intestinal fluid containing 20 mg/ml G-PUR®. After a 2-hour incubation at room temperature, bound and unbound deoxycholate were separated by centrifugation and the free radioactivity was quantified by liquid scintillation. Data obtained from 3 independent experiments are shown as means ± standard deviations.

Bile acids, including their bacterial breakdown products such as deoxycholate, are physiological constituents of the intestinal lumen. There is evidence for their binding to clinopitlolite [41]. Hence their potential to interfere with the binding of *C*. *difficile* toxins A and B to G-PUR® at low concentrations (2.5 and 0.25 mM) was tested. G-PUR® retained its ability to bind C. *difficile* toxins A and B in the presence of sodium deoxycholate or bile salts (Fig 3A). Conversely, binding of radiolabelled [^3^H]deoxycholate to G-PUR® was also examined: binding of [^3^H]deoxycholate was saturable; the fit of the data point to a rectangular hyperbola yielded estimates (± S.E.) for K_D_ = 5.95 ± 0.67 μM and for the binding capacity B_max_ = 3.1 ± 1.3 nmol/10 mg,corresponding to 160 μg/g) (Fig 3B).

### Prevention of *C*. *difficile* toxin-induced glucosylation of RAC1 by G-PUR®

**Fig 4:**
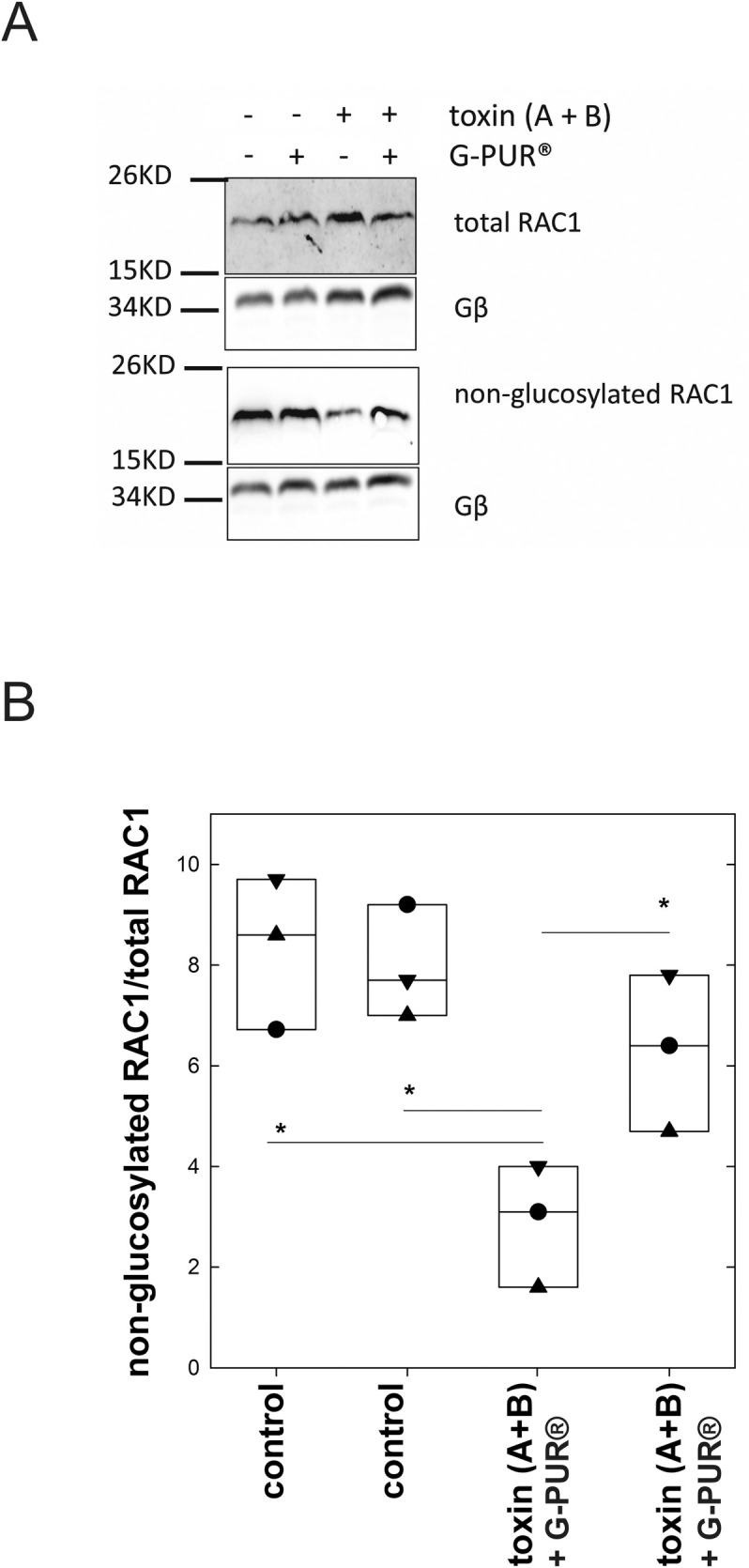
Quantification of RAC1 glucosylation in cellular lysates of Caco-2 cells incubated in the absence and presence of clostridial toxins by immunoblotting. Upon incubation in the absence and presence of *Clostridioides difficile* toxins A and B (0.2 ng/ml, equimolar mixture), Caco-2 cells were lysed and semi-quantification of RAC1 glucosylation was performed on immunoblots using monoclonal antibodies, which either recognize all forms of RAC1 (mAb23A8) or solely its non-glucosylated form (mAb102). G protein β-subunits (G-β) were visualized as a loading control. Panel A shows one representative western blot taken from 3 independent experiments. B. Data from three independent experiments are displayed using one distinctive symbol for each experiment. The median and interquartile range is also indicated by the box plot. The statistical comparison was done by repeated measures ANOVA followed by pairwise comparisons using the Holm-Sidak approach. *: significant difference at p<0.05. Original uncropped images of blots are shown in the S1 Raw images file.

The cellular substrates of *C*. *difficile* toxins A and B are small G proteins of the RHO-family, that is, RHO, RAC and CDC42. These are modified by O-linked monoglucosylation on a threonine residue (T37 in RHO and T35 in RAC and CDC42) in the effector loop, which leads to their inactivation [6, 15]. Monoglucosylation of RAC1 can be monitored by the differential reactivity of the protein to monoclonal antibodies (mAbs): mAb102 does not recognize glucosylated RAC1; in contrast, mAb23A8 detects all forms of RAC1 [36]. Accordingly, we incubated polarized monolayers of Caco-2 cells with an equimolar mixture of *C*. *difficile* toxins A and B, pre-incubated or not with G-PUR®, for 20 h. The selected toxin concentration (0.2 ng/ml) was low enough to preclude cell death. Incubation with toxins resulted in loss of immunoreactivity to the mAb102 (lane 3, lower set of blots in Fig 4A) but not to mAb23A8 (lane 3, upper set of blots in Fig 4A). In contrast, there was no loss of RAC1 immunoreactivity to mAb102, when the toxins had been pre-incubated with G-PUR® (lane 4, lower set of blots in Fig 4A). The sole addition of medium, which had been exposed to G-PUR®, did not affect RAC1 glucosylation (lane 2, lower set of blots in Fig 4A). The bands of immunoblots obtained from three independent experiments were quantified by densitometry (Fig 4B). The levels of non-glucosylated RAC1 in cells which had been treated with toxin A and B were significantly lower than in control cells and in cells treated with toxins containing medium preabsorbed to G-PUR® (p = 0.004 in both instances; Fig 4B). They were also significantly different from those seen after exposure to toxin-free medium preabsorbed to G-PUR® (p = 0.016; Fig 4B).

### Prevention of *C*. *difficile* toxin-induced microvillar disorganization by G-PUR®

**Fig 5:**
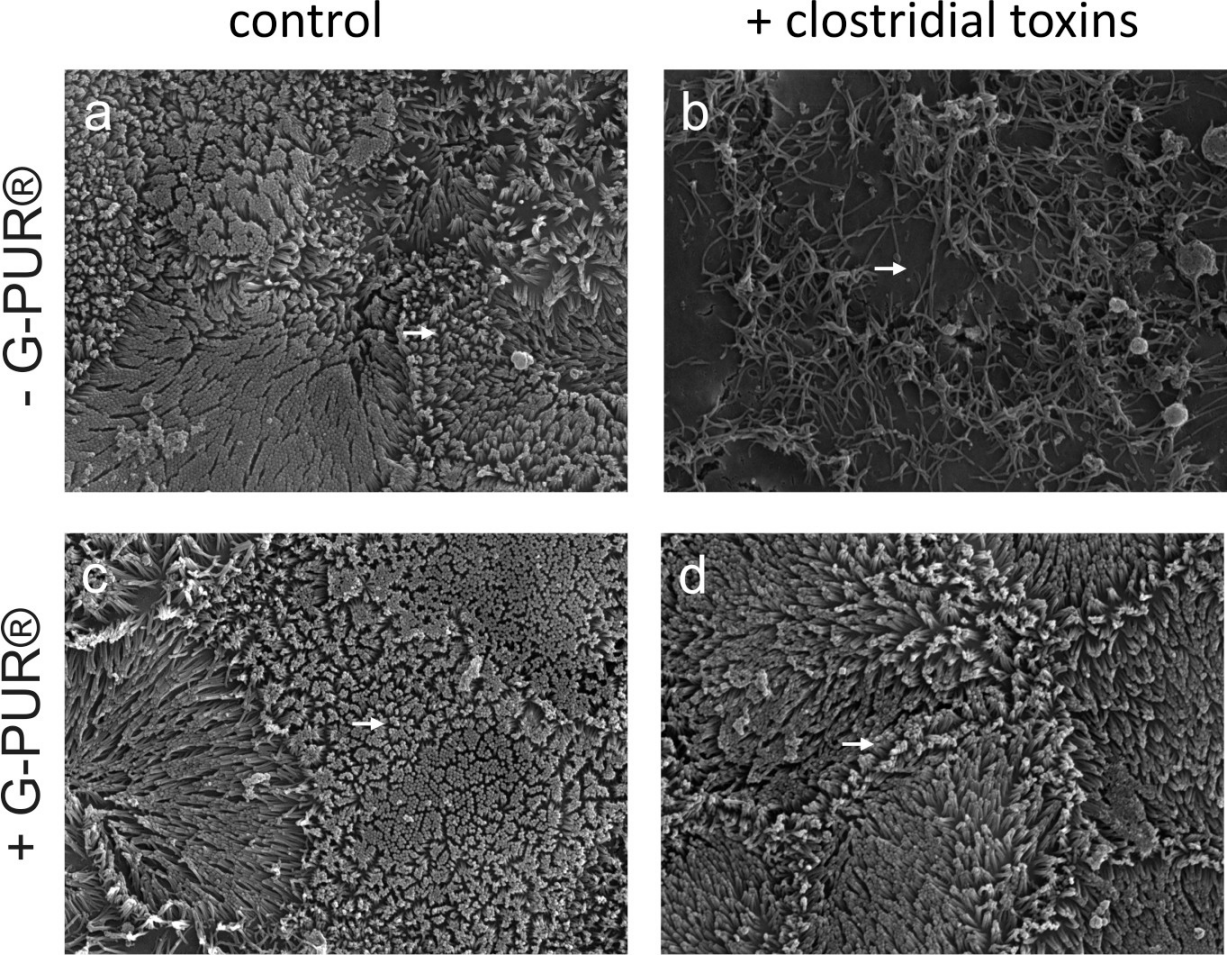
Scanning electron microscopy of the apical surface of Caco-2 cell monolayers incubated in the absence and presence of clostridial toxins. Monolayers of Caco-2 cells were exposed to medium (a,c; control) or medium containing an equimolar mixture (2.0 ng/ml) of *Clostridioides difficile* toxins A and B (b,d). The medium and the medium containing toxins were pre-adsorbed to G-PUR® (4 mg/ml) for 15 min in panels c and d, respectively. Images were taken at 23,000-fold magnification. For each sample, a minimum of 1000 cells was assessed. Arrows point to microvilli and show that non-treated toxin mix (b) disorganized the brush border whereas toxin mix pre-treated with G-PUR® completely restored the microvillar organization (d).

The organization of the actin cytoskeleton depends on functional small G proteins of the RHO-family. Formation and maintenance of microvilli in the intestinal epithelium relies on a core actin bundle [42] and requires the activity of RAC1. In fact, the inducible deletion of RAC1 in intestinal epithelial cells abrogates microvilli [43]. The data summarized in Figs 2 and 3 indicate that G-PUR® bound *C*. *difficile* toxins A and B and precluded their uptake by Caco-2 cells thus preventing the inactivation of RAC1 in Caco-2 cells. We verified that this translated into a preserved microvillar brush border by incubating polarized monolayers of Caco-2 cells with *C*. *difficile* toxins prior to and after their adsorption to G-PUR®. Scanning electron microscopy visualized abundant microvilli on the apical surface of polarized Caco-2 cells (Fig 5A). Their density was not affected if the medium was pretreated with G-PUR® (Fig 5C). In contrast, if the monolayer was incubated with an equimolar mixture of *C*. *difficile* toxins A and B (2 ng/ml) for 5 h, the brush border was severely affected: the apical surface of the cells was only covered with sparse, loosely distributed protrusions (Fig 5B). Pre-adsorption of the toxins onto G-PUR® completely prevented the loss of microvilli such that the brush border was morphologically indistinguishable from that of control cells (Fig 5D).

### Preservation of cellular junctions and of transepithelial electrical resistance (TEER) by adsorption of *C*. *difficile* toxins to G-PUR®

**Fig 6:**
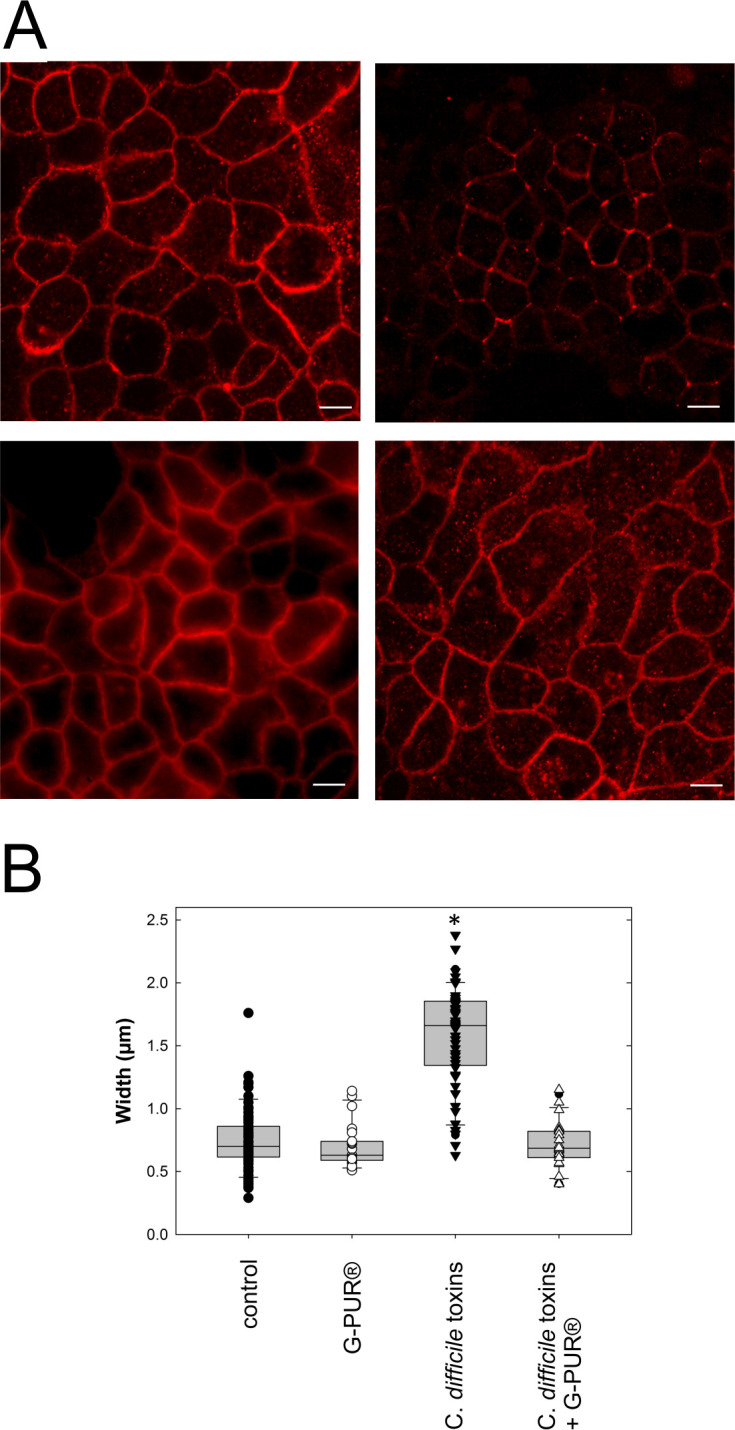
Apical occludin staining pattern and intensity in Caco-2 cell monolayers incubated in the absence and presence of clostridial toxins. A. Representative images of the apical surface of Caco-2 cell monolayers incubated in the absence (upper left panel) and presence (upper right panel) of an equimolar mixture (2.2 ng/ml) of *Clostridioides difficile* toxins A and B. The lower left panel shows the occludin staining obtained with cells incubated with a toxin-free medium pre-treated with G-PUR®. In the lower right panel, the toxins were adsorbed onto G-PUR® (4 mg/ml) for 15 min prior to addition to the culture medium. Images are representative of 20 visual fields/condition in an experiment carried out in parallel. A second experiment gave similar results. The scale bars correspond to 10 μm. B. Redistribution of occludin staining was quantified by measuring the width of the occluding membrane staining in a minimum of 50 cellular contacts per condition. Cells treated with the equimolar mixture (2.2 ng/ml) of *Clostridioides difficile* toxins A and B differed from all other conditions by the more widely distributed occludin on their membranes (*, p<0.05 Kruskal-Wallis test followed by Dunn’s multiple comparisons). In contrast, all other pairwise comparisons did not provide any evidence for a difference.

The hallmark of CDI is acute diarrhea resulting from a disruption of the intestinal barrier due to the action of the *C*. *difficile* toxins A and B on enterocytes. At the cellular level, this is associated with a disorganization of the F-actin ring, which maintains the integrity of the tight junctions [17, 44]. The ensuing disorganization of the intercellular junctions results in redistribution of protein constituents of the tight junctions, among which are ZO-1 and occludin [45]. We recapitulated these findings by incubating monolayers of Caco-2 cells with an equimolar mixture of toxin A and B (2.2 ng/ml) for 24 h followed by immunostaining for occludin. In control cells, occludin staining revealed continuous labeling of the apical cellular membranes (Fig 6A, upper left panel). In contrast, in toxin-treated cells, occludin staining of the apical membrane was reduced and the distribution was inhomogeneous (Fig 6A, upper right panel). The effect of the *C*. *difficile* toxins was abolished, if they had been pre-adsorbed to G-PUR® (Fig 6A, lower right panel). Similarly, there wasn’t any appreciable morphological change in monolayers exposed to toxin-free culture medium, which had been pre-adsorbed to G-PUR® (Fig 6A, lower left panel). Cells exposed to the equimolar mixture of toxin A and B showed less variability in cell shape. Compared with the classical polygonals shape seen under control conditions (Fig 6A, upper left panel), they appeared rounder and, consequently, smaller (Fig 6A, upper right panel). This can be attributed to the action of the toxins.

Redistribution of occludin was quantified by measuring the width of occludin membrane distribution. Incubation of the Caco-2 cell monolayer with an equimolar mixture of toxin A and B resulted in an increased spreading of occludin staining (Fig 6B). The difference to all other conditions was statistically signifcant (p<0.05, Krukal-Wallis test followed by Dunn’s posthoc multiple comparisons). In contrast, pairwise comparisons of all other conditions did not reveal any appreciable difference.

**Fig 7:**
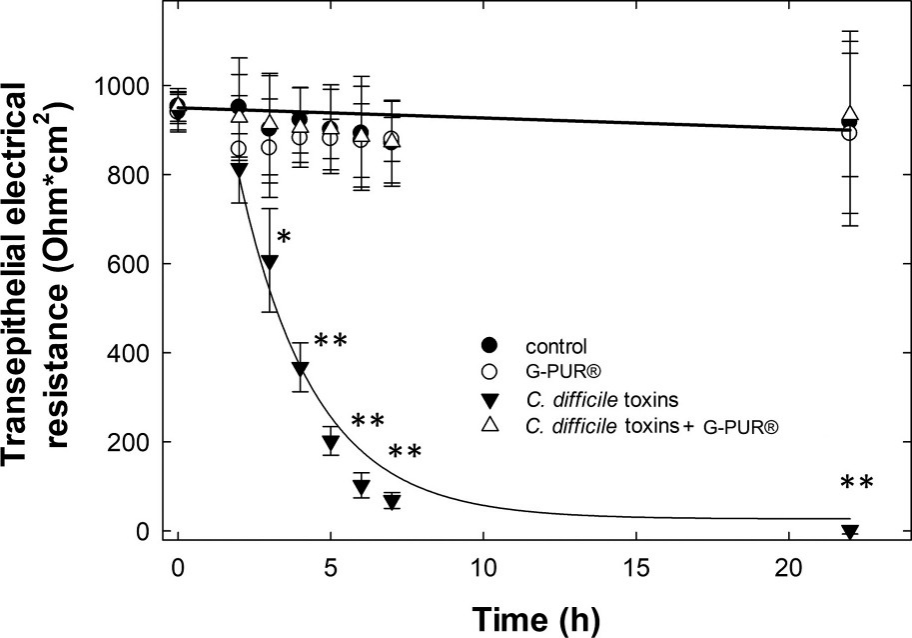
Change in transepithelial electrical resistance (TEER) of Caco-2 cell monolayers incubated with clostridial toxins. Caco-2 cell monolayers grown on Transwell™ inserts were incubated in the absence (●, ○) and presence of an equimolar mixture (2.5 ng/ml) of *Clostridioides difficile* toxins A and B (▼, Δ) at 37°C. Both types of media were either pre-adsorbed onto G-PUR® (4 mg/ml) for 15 min (○, Δ) or incubated for the same duration with medium (●,▼). At the indicated time points, the medium was replaced by fresh medium and the transepithelial electrical resistance (TEER) was measured. Data, represented by means ± SD from 3 independent experiments, showed that incubation with an untreated toxin mix lead to complete breakdown of the intestinal barrier whereas pre-incubation of the toxin mix preserved the electrical resistance of the intestinal model. The breakdown in electrical resistance progressed over a time course of hours with a time course, which was reasonably well described by a monoexponential decay (solid line of Fig 7). Three hours after addition of *Clostridioides difficile* toxins A and B (▼), the TEER of the Caco-2 monolayer was significantly lower than that of any other condition (* p = 0.013 versus clinoliptolite-absorbed medium and p = 0.007 versus both, control and clinoptilolite-absorbed toxins). From 4 h to 22 h, the statistiscal comparison showed differences with p<0.001 (**) for all conditions compared to toxin-treated monolayers. The statistical testing was done by repeated measures ANOVA followed by all possible pairwise comparisons using the Holm-Sidak approach with a threshold of significance at p<0.05.

These observations predict that G-PUR® may prevent the breakdown of the epithelial barrier induced by *C*. *difficile* toxins. We verified this prediction by monitoring the transepithelial electrical resistance of a Caco-2 monolayer. Caco-2 cells differentiated on a porous membrane form a tight monolayer, which displays a large resistance to rectangular currents delivered through chopstick electrodes (Fig 7). Pre-adsorption of the toxins-free medium to G-PUR® did not affect the ohmic resistance of the monolayer (Fig 7). As expected, addition of *C*. *difficile* toxins A and B led to a rapid loss of electrical resistance, which became statistically significant after 3 h (repeated measures ANOVA followed by all pairwise comparisons using the Holm-Sidak test). After 24 h, there was a complete breakdown of the epithelial barrier function (Fig 7). In contrast, the epithelial barrier was maintained and the electrical resistance of the monolayer was comparable to that seen under control conditions if the *C*. *difficile* toxins were pre-adsorbed to G-PUR® (Fig 7). We also confirmed by ELISA that the pre-adsorption had effectively removed the toxins: they were undetectable in the culture medium (S1 File).

## Discussion

Clinoptilolite has been used for many decades as a feed additive in animal husbandry [27]; its growth promoting effect is thought to arise–at least in part–from binding of mycotoxins [29]. Livestock are fed clinoptilolite for months to years without obvious adverse events [46, 47]. Hence, it is safe to posit that clinoptilolite is innocuous, in particular if contaminating heavy metals have been removed from its crystal lattice [48]. Our experiments showed that the purified clinoptilolite-tuff preparation G-PUR® bound *C*. *difficile* toxins A and B with high affinity. Binding those effectively precluded their cytotoxic actions on Caco-2 cell monolayers. This conclusion is based on the following lines of evidence: (i) toxin-induced glucosylation of RAC1 was blocked in Caco-2 cells, indicating that the primary cellular targets, the small G proteins of the RHO family, were preserved. (ii) Disorganisation of the actin cytoskeleton, as reflected by the distortion of microvilli at the brush border and apical denudation of Caco-2 cells could be prevented by G-PUR®.(iii) Similarly, because *C*. *difficile* toxins abrogate the G protein-dependent regulation of the cytoskeleton, the apical F-actin ring is dissolved and tight junctions are dismantled [17, 45]. We visualized this action of *C*. *difficile* toxins by staining for occludin: binding of the toxins to G-PUR® abrogated their effect on apical occludin distribution. (iv) Loosening of tight junctions leads to breakdown of the epithelial barrier [45] and promotes the flux of water and solutes, leading to diarrhea [17]. The barrier function was assessed by recording transepithelial electrical resistance (TEER) of the Caco-2 cell monolayer: adsorption of *C*. *difficile* toxins A and B onto G-PUR® prevented the decline in TEER.

We are aware of the fact that our study has limitations, because CDI manifests itself in the colon and the Caco-2 model recapitulates many features of the small intestine. Nevertheless, this human intestinal model has been extensively used over the last decades to study intestinal permeability. In fact, it was already implemented to investigate the colonization and dissemination mechanisms of C. *difficile* [49, 50]. It has also been used as a cell culture model to investigate the restoration of the disrupted intestinal barrier by cannabidiol after exposure to C. *difficile* toxin A [51]. In our binding assay, we observed biphasic adsorption of *C*. *difficile* toxins to G-PUR®: after an initial rapid phase (apparent on rate ~0.3 min^-1^), which accounted for about 50% of the adsorption, binding proceeded at a roughly 20-fold slower rate (apparent on rate ~0.016 min^-1^). The mechanism underlying this biphasic nature is not clear. It can be rationalized by assuming that, because of their size (about 300 kDa), toxins can at first only bind to sites on the surface of the particles. Binding sites within the microporous structure are reached more slowly.

*C*. *difficile* toxins are very potent; concentrations in the picomolar range suffice to trigger a breakdown of the epithelial barrier [52]. In fact, fecal concentrations were found to range from 3 to 50 ng/ml, which corresponds to about 10 to 170 pM, for mild (grade 2) to moderate to severe (grade 5) disease [53]. We observed half-maximum saturation of binding to G-PUR® at a concentration of *C*. *difficile* toxins of 23.7 ng/ml (about 80 pM). Thus, the affinity of clinoptilolite for *C*. *difficile* toxins matches their affinity for their biological targets.

Bile acids including deoxycholate are present in the intestinal lumen. Previous studies provided evidence for adsorption of bile acids to various forms of clinoptilolite [41, 54]. Accordingly, we directly examined the binding of radioactively labeled deoxycholate to G-PUR®. The experiments showed that G-PUR® bound deoxycholate with surprisingly high affinity. More importantly, we verified that bile salts and deoxycholate up to a concentration of 2.5 mM did not interfere with binding of clostridial toxins to G-PUR®.

G-PUR® bound about 10 ng *C*. *difficile* toxins per mg in vitro. This binding capacity is comparable or slightly superior to previously published results for tolevamer [24, 55]. When used as dietary supplement at a daily dose of 2 g, the predicted adsorption can amount to up to 20–40 μg of *C*. *difficile* toxins, therefore this may be expected to suffice to neutralize the toxins present in mild disease assuming 3 μg/l intestinal fluid [53].

Our experiments indicate that higher doses of G-PUR® may also be safely administered: the total surface of the human digestive tract has been estimated to be about 32 m² [56]. Accordingly, a dose of 2 g G-PUR® translates into an average deposition of 6.2 μg G-PUR® per cm² along the digestive tract. We exposed a Caco-2 cell monolayer to a density of 200 μg/cm² for 24 h and did not observe any signs of toxicity by assessing their metabolic activity and by quantifying the amount of live and dead cells. Similarly, we did not detect any morphological changes. Thus based on these considerations and previous observations showing the innocuousness of the oral administration of G-PUR [48], we conclude that the safety margin of G-PUR® is large.

Antibiotics and antibacterial agents are effective in the management of CDI, but recurrent diarrhea is frequent [3, 23]. In addition, fulminant colitis is poorly responsive to antibiotic treatment [57]. Accordingly, toxin binding by monoclonal antibodies [58] or by anion [20, 21] and cation exchange resins [25] have been explored as an adjunct or as an alternative. Recurrences are thought to arise from newly ingested spores and from persistent dysbiosis: the alterations in the gut microbiome, which promote the emergence of CDAD, require several weeks for their correction [3, 23]. Dysbiosis is less likely to occur with toxin binders: in fact, tolevamer was inferior to vancomycin or metronidazole with respect to curing diarrhea but superior with respect to recurrent disease [25]. It is not clear how toxin binding resins prevent recurrences, but bile acid sequestration may be important: bile acids (cholic acid and deoxycholic acid) act as germinants in conjunction with glycine and promote the germination of *C*. *difficile* [59, 60]. Bile acids also provide a link to the microbiome: deoxycholic acid, a product of bacterial metabolism in the colon, inhibits growth of *C*. *difficile* [59]. Thus, the normal colonic microbiome may restrict the proliferation of *C*. *difficile* by supplying deoxycholate [59]. It has been appreciated some 30 years ago that recurrent *C*. *difficile*-induced diarrhea can be remedied by administering a mixture of bacteria including *Bacteroides spp*. [61]. This was confirmed in a randomized trial, where a suspension of donor feces was administered to patients suffering from *C*. *difficile*-induced diarrhea [62]. Restoration of *Bacteroidetes* is an important component in the recovery of the microbiome and is associated with protection against recurrence of *C*. *difficile*-induced diarrhea [63]. As shown in the present study and earlier work, clinoptilolite binds bile acids [41, 54].

Taken together, the present findings and our earlier work [48] show that appropriately purified preparations of clinoptilolite-tuff (G-PUR®) have three interesting properties, which are relevant to the pathophysiology underlying recurrent *C*. *difficile*-induced diarrhea: they adsorb *C*. *difficile* toxins A and B and neutralize their biological activity, they bind bile acids and they may promote the restoration of the gut microbiome. It is not clear, if G-PUR® will be superior to vancomycin in curing acute *C*. *difficile*-induced diarrhea. However, it is reasonable to assume that G-PUR® has the potential to prevent recurrent disease. We propose that this conjecture is worthwhile exploring.

## Supporting information

S1 Raw images This file contains the original, unprocessed images of the western blots presented in Fig 4. (TIF)

S1 File Quantification of C. *difficile* toxins A and B in a cell culture medium upon incubation of Caco-2 intestinal cells with a toxin mix pre-treated with G-PUR®. (DOCX)
