# Supplementary figures and images for: Binding and neutralization of C. difficile toxins A and B by purified clinoptilolite-tuff
Source: PLoS One. 2021 May 27;16(5):e0252211. doi: 10.1371/journal.pone.0252211 (PMC8158989; doi:10.1371/journal.pone.0252211)

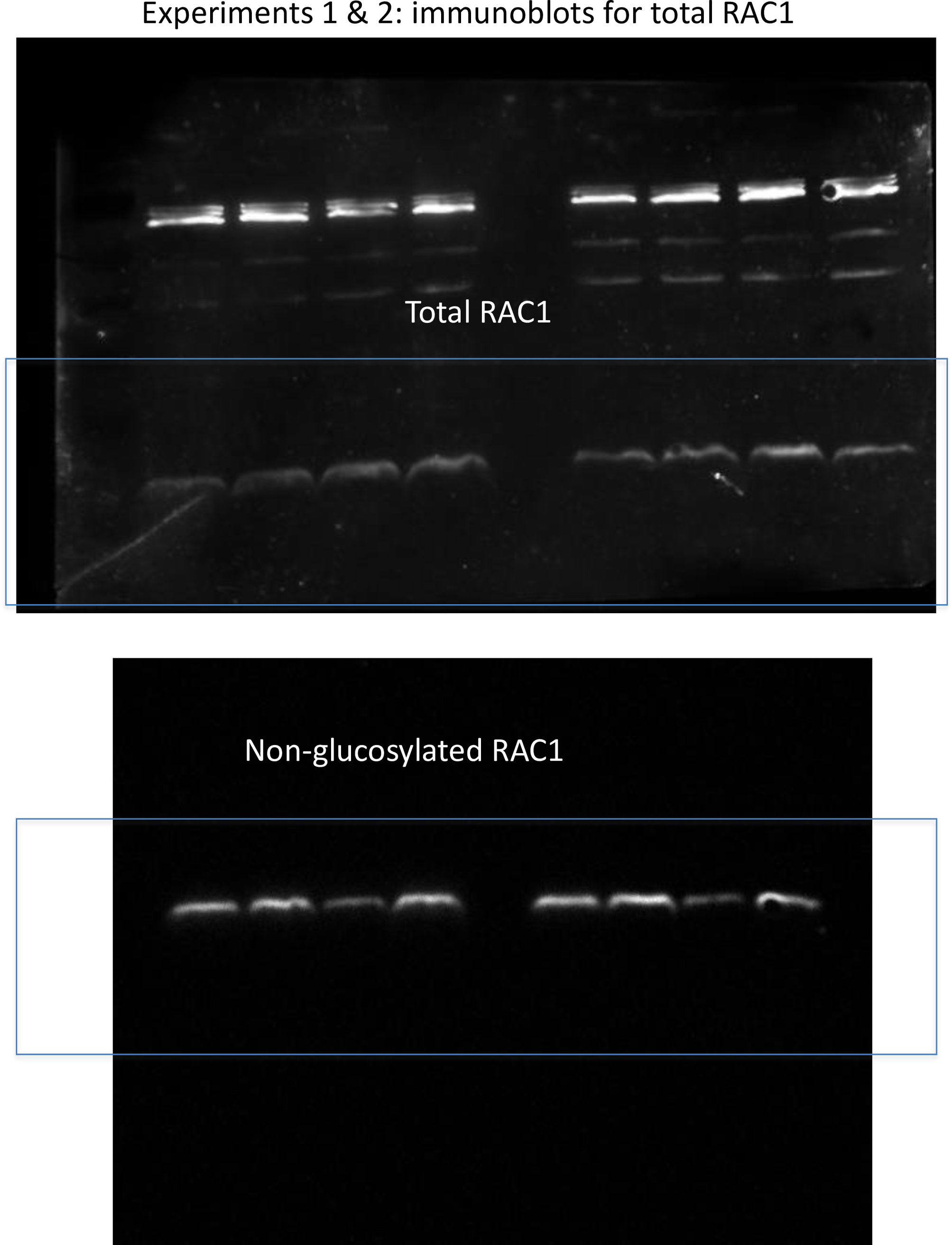

Supplement: S1 Raw images — (TIF) [file pone.0252211.s001.tif]
