# Supplementary material for: Binding and neutralization of C. difficile toxins A and B by purified clinoptilolite-tuff
Source: PLoS One. 2021 May 27;16(5):e0252211. doi: 10.1371/journal.pone.0252211 (PMC8158989; doi:10.1371/journal.pone.0252211)
Supplement: S1 File — (DOCX) [file pone.0252211.s002.docx]

**S1 File. Quantification of C. *difficile* toxins A and B in cell culture medium of Caco-2 intestinal cells incubated with a toxin mix pre-treated with G-PUR®**

|  | Starting concentration [ng/ml] | | End concentration without G-PUR® [ng/ml] | | End concentration with G-PUR® [ng/ml] | | Limit of quantification [ng/ml] |
| --- | --- | --- | --- | --- | --- | --- | --- |
|  | mean | SD | mean | SD | mean | SD |  |
| Exp 1 | 2.71 | 0.10 | 2.30 | 0.08 | 0.05 | 0.00 | 0.2 |
| Exp 2 | 2.58 | 0 | 2.08 | 0.14 | 0.09 | 0.00 | 0.2 |
